# Author Correction: Synergistic effects of combinatorial chitosan and polyphenol biomolecules on enhanced antibacterial activity of biofunctionalized silver nanoparticles

**DOI:** 10.1038/s41598-021-84489-y

**Published:** 2021-02-23

**Authors:** Niloufar Hajarian Rezazadeh, Foad Buazar, Soheila Matroodi

**Affiliations:** 1 Department of Marine Chemistry, Khorramshahr University of Marine Science and Technology, PO. Box 669, Khorramshahr, Iran; 2 Department of Marine Biology, Khorramshahr University of Marine Science and Technology, PO. Box 669, Khorramshahr, Iran

Correction to: *Scientific Reports* 10.1038/s41598-020-76726-7, published online 12 November 2020

The original version of this Article contained an error in the title of the paper, where the word “biofunctionalized” was incorrectly given as “biofunctionalaized”. This has now been corrected in the PDF and HTML versions of the Article.

